# Real-world Use of Molecular Point-of-care Testing for Sexually Transmitted Infections (STIs) in the Emergency Department: Why It Matters for Acute Care Management

**DOI:** 10.1093/ofid/ofaf749

**Published:** 2025-12-12

**Authors:** Gaby Dashler, Kendall Maliszewski, Mustapha Saheed, Edana Mann, Nyah Johnson, Spencer J Mann, Tracy Colburn, William Clarke, Charlotte A Gaydos, Yukari C Manabe, K Davina Frick, Richard E Rothman, Yu-Hsiang Hsieh

**Affiliations:** Department of Emergency Medicine, School of Medicine, Johns Hopkins University, Baltimore, Maryland, USA; Department of Emergency Medicine, School of Medicine, Johns Hopkins University, Baltimore, Maryland, USA; Department of Emergency Medicine, School of Medicine, Johns Hopkins University, Baltimore, Maryland, USA; Department of Emergency Medicine, School of Medicine, Johns Hopkins University, Baltimore, Maryland, USA; Department of Emergency Medicine, School of Medicine, Johns Hopkins University, Baltimore, Maryland, USA; Department of Emergency Medicine, School of Medicine, Johns Hopkins University, Baltimore, Maryland, USA; Department of Emergency Medicine, School of Medicine, Johns Hopkins University, Baltimore, Maryland, USA; Department of Pathology, Johns Hopkins University School of Medicine, Baltimore, Maryland, USA; Department of Medicine, Division of Infectious Diseases, Johns Hopkins University School of Medicine, Baltimore, Maryland, USA; Department of Medicine, Division of Infectious Diseases, Johns Hopkins University School of Medicine, Baltimore, Maryland, USA; Department of Operations Management and Business Analytics, Carey Business School, Johns Hopkins University, Baltimore, Maryland, USA; Department of Emergency Medicine, School of Medicine, Johns Hopkins University, Baltimore, Maryland, USA; Department of Medicine, Division of Infectious Diseases, Johns Hopkins University School of Medicine, Baltimore, Maryland, USA; Department of Emergency Medicine, School of Medicine, Johns Hopkins University, Baltimore, Maryland, USA

**Keywords:** antibiotic over- and under-treatment, emergency department, emergency department length of stay, molecular point-of-care test, sexually transmitted infections (STIs)

## Abstract

**Background:**

Point-of-care (POC) polymerase chain reaction (PCR) tests for sexually transmitted infections (STIs) represent a potential paradigm shift for emergency department (ED) management of patients with suspected STIs, given there are now Food and Drug Administration–cleared POC tests that permit definite rapid diagnosis and result-driven care.

**Methods:**

A quasi-experimental real-world implementation study was conducted in an urban ED, comparing two approaches for female STI testing for *Chlamydia trachomatis* (CT), *Neisseria gonorrhoeae* (NG), and *Trichomonas vaginalis* (TV): (1) central laboratory testing (August–November 2022) with batched nucleic acid amplification testing (CT/NG) and wet prep for TV; (2) POC PCR Testing Integration (ED POC) (January–April 2023) in an ED POC laboratory for all three STIs. We compared proportions of appropriate treatment and ED length of stay (LOS) between the two testing modalities using chi-square test and log-transformed multivariable linear regression, respectively.

**Results:**

Of 627 patients, 340 received central laboratory testing and 287 received ED POC; ED POC resulted in a significant decrease in LOS by 76 minutes or 9.3% (95% confidence interval [CI], −16.3% to −1.7%; *P* = .017). ED POC also significantly lowered overtreatment rates for CT (n = 595) and NG (n = 607) by 73% (95% CI, 44–87; *P* < .001) and 63% (95% CI, 28–81; *P* = .002), respectively. ED POC testing was associated with 67% lower rate of undertreatment (95% CI, −19% to 91%; *P* = .093) for any CT/NG/TV-positive (n = 78), but not statistically significant due to relatively small number of undertreated cases .

**Discussion:**

Compared to traditional STI testing, POC PCR testing significantly shortened ED LOS, allowed for organism-specific targeted treatment, and reduced overtreatment of CT and NG infections.

## Teaser

This study implemented and evaluated a point-of-care (POC) test for sexually transmitted infections (STIs) in a large, academic emergency department. We found the POC test significantly reduced overall length of stay and overtreatment of suspected infections for patients who received STI testing.

In the United States, approximately 140 million individuals visit emergency departments (EDs) annually [1], with disproportionately higher numbers of visits for individuals facing socioeconomic and health disparities. These individuals are more vulnerable to the most commonly diagnosed nonviral sexually transmitted infections (STIs) including *Chlamydia trachomatis* (CT), *Neisseria gonorrhoeae* (NG), and *Trichomonas vaginalis* (TV) [2–4]. In recent years, STI-related complaints and diagnoses in EDs have significantly increased [5, 6]. Routine ED practice relies on syndromic diagnosis of STIs and presumptive antimicrobial treatment based on symptoms and risk factors, rather than confirmed laboratory results [7, 8]. This approach has notable shortcomings, including inadequate reduction of community transmission and rising rates of antimicrobial resistance due to undertreatment and overtreatment, respectively [9–11]. A recent scoping review highlights these shortcomings, with reported CT/NG positivity rates up to 52% among patients who were not empirically treated in the ED, and as low as 14% among those presumptively treated [7].

One of the primary reasons ED clinicians and current practice guidelines continue to rely on empiric antimicrobial STI treatment is the inability to obtain timely molecular test results during the clinical encounter [12]. Nucleic acid amplification tests (NAATs) for CT, NG, and TV have revolutionized the landscape of STI testing; however, turnaround times for NAAT results generally remain 1 to 2 business days, likely due in part to their handling in central laboratories where skilled laboratory technicians perform the assays in batches [9].

Over the past decade, rapid point-of-care (POC) assays, which can be done near the bedside by nonlaboratory users (ie, Clinical Laboratory Improvement Amendments [CLIA]-waived) and provide a definitive diagnosis and appropriate treatment in a single visit [13], have been approved by the US Food and Drug Administration (FDA) [14]. Results from our previous randomized controlled trial, which utilized research coordinators to perform the Cepheid CT/NG Xpert rapid PCR test for detecting CT and NG from provider-collected endocervical swabs, indicated a significant reduction in both undertreatment and overtreatment of STIs. This improvement was achieved with a median turnaround time of 104 minutes, but in a well-controlled research setting [9]. Similar findings on use of Xpert rapid polymerase chain reaction (PCR) test in EDs were observed in one smaller randomized controlled trial, two quasi-experiment studies, and one before-and-after implementation study [15–18], as well as one retrospective cohort study that used Binx IO [19]. Notably, impediments with routine POC testing hinder widespread adoption, including higher per-sample costs and the need to establish ED POC workflows. We sought to explore these challenges and pilot molecular POC STI testing in the ED.

In 2021, the FDA cleared the Visby Medical Sexual Health Test (Visby Medical, San Jose, CA, USA)—a rapid, single-use, highly accurate multiplex POC PCR assay for detecting CT, NG, and TV on vaginal swabs—as a CLIA-waived diagnostic test for clinical use for female patients [20]. We piloted a program incorporating this POC test into our ED to evaluate the clinical impact of rapid POC testing for CT, NG, and TV (operationally defined going forward as the “study STIs”). We compared it to use of standard central laboratory testing for female ED patients. The primary objectives were to assess the proportion of patients receiving appropriate treatment (ie, rates of under- and overtreatment) and to evaluate differences in specific ED workflow times between the groups, including overall length of stay (LOS).

## METHODS

### Study Design

A quasi before-and-after pilot implementation study was conducted at the Johns Hopkins Hospital ED (JHHED), comparing two approaches for STI testing in female patients; (1) ED performed POC PCR testing (ED POC), in which an ED POC test for study STIs was completed utilizing the Visby Medical Sexual Health Test, versus (2) central laboratory–performed NAAT and wet mount preparation (wet prep) testing (Central), in which the central laboratory tested for CT/NG with NAAT, and for TV with wet prep and/or NAAT based on the provider's order.

The study periods were selected from 1 August to 30 November 2022 (Central-only phase) when only Central operated, followed by the period when POC PCR testing was integrated with ED workflow from 9 January to 28 April 2023 (ED POC integration phase). The period we selected as reference was August-November 2022 (instead of January-April) due to marked increases in COVID-19 rates (omicron variant) in our ED [21, 22], which significantly increased the ED LOS, one of our main outcomes. We educated providers to select POC testing as the preferred testing method during the POC implementation period but made NAAT available per provider's discretion.

The study was approved by The Johns Hopkins University School of Medicine Institutional Review Board (IRB00316241) and conducted in accordance with the ethical standards of the Helsinki Declaration of the World Medical Association.

### Study Setting

The JHHED, an urban academic adult ED in Baltimore, Maryland, provides services to approximately 60 000 patients annually. Historically, a relatively high prevalence was reported for each of CT (7.9%–9.3%) and NG (3.9%–5.3%) in female patients, based on screening program data in 1998 and a clinical trial in 2015–2016, respectively [9, 23]. The TV-positive rate in 2014–2015 was 19.1% [24]. In 2020, the JHHED established an ED POC laboratory that serves as a clinical space to operate CLIA-waived tests, first functioning for POC SARS-CoV-2 testing. In the JHHED, STI testing is ordered at the clinician's discretion in accordance with departmental protocols that adhere to the latest U.S. Centers for Disease Control and Prevention’s (CDC) *Sexually Transmitted Infections Treatment Guidelines* [25]. These CDC-recommended protocols are embedded within the electronic medical records (EMR) for real-time reference.

## INTERVENTIONS

### STI Testing

Standard NAAT STI testing during both central only and ED POC integration phases were performed daily in batch (Roche cobas 6800 CT/NG assay and Roche cobas 6800 TV/MG assay) on urine and vaginal and cervical swab specimens. Wet prep testing for TV, which has a reported sensitivity of approximately 40%–60% and a specificity of 99%–100% when compared with NAAT [24], was performed on a vaginal swab that was transported directly to the central microbiology laboratory. The turnaround time for results was approximately 30 minutes from specimen collection.

**Figure 1.**
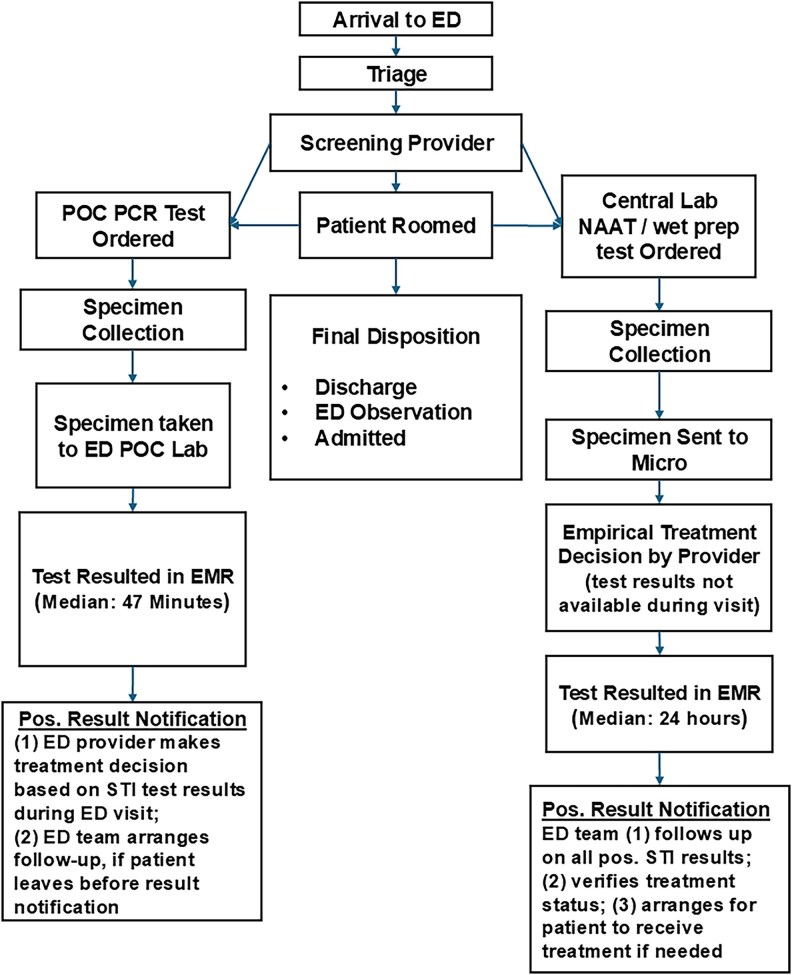
Clinical workflow for sexually transmitted infection testing by testing modality at the Johns Hopkins Hospital Adult Emergency Department during the study period.

POC PCR STI testing was integrated into ED workflows under the guidance of ED clinical administration with support from Department of Pathology (Figure 1). A detailed description is presented in the Supplementary Methods Section. For POC testing, the patient self-collected a vaginal swab specimen or the provider collected swabs during a pelvic examination. The specimen was immediately taken to the ED POC laboratory and trained nursing assistants performed the test following the manufacturer's operational guidelines.

### Study Participants

Adult female patients who received testing for CT and NG were included, either with or without testing for TV. Patients with any of the following characteristics were excluded from analysis: transgender women, left without being seen, left against medical advice, eloped, or transferred to other facilities. We also restricted inclusion to those patients who received STI testing order as part of their primary diagnostic workup; patients who had STI testing after they were admitted to an observation or inpatient unit were excluded. If a patient had multiple visits with STI testing during the study period, only the first visit was included for analysis.

### Outcomes

The primary outcome measures included (1) the proportion of patients with a laboratory-confirmed study STI test who were treated (ie, given antibiotics in the ED) or prescribed (sent home with a prescription) appropriate antibiotics during their ED stay and (2) ED LOS. Appropriate treatment included antibiotics administered in the ED or prescribed at discharge according to 2021 CDC STI Treatment Guidelines (doxycycline or azithromycin for positive CT results, ceftriaxone for positive NG results, and metronidazole for positive TV results) [25]. Overtreatment was operationally defined as a patient with a negative STI test who was treated with or prescribed antibiotics during their ED visit. Undertreatment was operationally defined as a patient with a positive STI test who was discharged from the ED without appropriate treatment or a prescription.

Secondary outcomes included: turnaround time for STI testing; proportion of patients with laboratory-confirmed study STIs; proportion of patients with STI results available during the ED visit; proportion of patients informed of their positive STI results; proportion of patients re-presenting to the ED within 30 days for STI-related chief complaints (ie, vaginal discharge, vaginal bleeding, lower abdominal pain, dysuria). “Informed of their positive results” was operationally defined as any patient with a positive STI result who received appropriate treatment in the ED, or with documentation of result notification in the EMR (ie, by phone call after discharge).

### Data Collection and Analysis

All data were collected using a standard data collection form by ED research coordinators via retrospective EMR review. Descriptive analysis was performed to summarize the categorical and continuous variables analyzed in the study. We categorized patients who received POC PCR testing with the Visby Medical Sexual Health Test as the ED POC group and those who received central laboratory NAAT only as the Central group. To mitigate confounders, patients who received STI testing during overnight hours when the ED POC laboratory was not staffed (12 am–9 am) were excluded from analysis.

Multivariable linear regression analysis was performed to estimate the association between POC PCR testing and the log-transformed time interval between ED arrival and departure time stamps (total ED LOS) as an outcome variable, with adjustment of covariates that had a *P* < 0.2 in the bivariate analysis. Stepwise variable selection for the multivariable regression analysis was used to estimate the adjusted percentage of time (from ED arrival to departure) decreased or increased by testing modality. Detailed descriptions of further analysis are provided in the Supplemental Methods. SAS version 9.4 software (SAS Institute, Cary, North Carolina) was used for all analyses and a 2-sided *P* < .05 was considered statistically significant.

## RESULTS

### Characteristics of Patients Included

During the study periods, a total of 627 patients received testing for study STIs, including 297 (47.4%) evaluated in the only phase and 330 (52.6%) in the ED POC integration phase. Of those 330 in the ED-POC integration phase, 287 (87%) received POC testing; 43 (13.0%) received central-based on provider placed order, resulting in 287 patients in the ED POC group and 340 in the Central group.

Sociodemographic characteristics of patients based on test modality and implementation phase are summarized in Table 1 and Supplementary Table 1, respectively. Overall, groups were similar except for pregnancy and HIV (Table 1). Those tested by POC were less likely to have had a pelvic examination (Central: 73.2% versus ED POC: 63.4%; *P* = .008). The leading symptom was lower abdominal pain (59.1%), followed by vaginal bleeding (27.7%), and vaginal discharge (25.9%). The pattern of presenting symptoms did not differ between the Central and ED POC groups. For those 340 patients in the Central group, there were no statistical differences in demographic characteristics in patients who were in Central only phase or in the ED POC integration phase (Supplementary Table 2).

**Table 1.** Sociodemographic and Clinical Characteristics Among 627 Female Johns Hopkins Hospital Adult Emergency Department Patients Who Received Testing for Chlamydia, Gonorrhea, and Trichomonas From 1 August 2022 to 30 November 2022 and From 9 January 2023 to 28 April 2023

		STI Testing Modality			*P* Value^b^
Characteristics	Category	Total	Central Lab Testing^a^	ED-POC Testing^a^	
Overall		627	340	287	
A. Sociodemographics					
Age (y)	Mean	31.7 ± 11.1	32.4 ± 11.8	30.9 ± 10.1	*.084*
	Median	30 [23, 36]	30 [24, 36]	30 [23, 37]	.221
	18–24	193	97 (28.5)	96 (33.5)	.200
	25–29	115	71 (20.9)	44 (15.3)	
	30–34	126	63 (18.5)	63 (22.0)	
	35–39	80	41 (12.1)	39 (13.6)	
	≥ 40	112	67 (19.7)	45 (15.7)	
	Missing	1	1 (0.3)	0 (0.0)	
Gender	Female	623	338 (99.4)	285 (99.3)	.779
	Transgender	3	1 (0.3)	2 (0.7)	
	Nonbinary	1	1 (0.3)	0 (0.0)	
Race/ethnicity	White, non-Hispanic	68	41 (12.1)	27 (9.4)	.735
	Black, non-Hispanic	403	215 (63.2)	188 (65.5)	
	Other race, non-Hispanic	54	28 (8.2)	26 (9.1)	
	Hispanic	102	56 (16.5)	46 (16.0)	
B. Clinical characteristics					
Self-reported	Yes	154	83 (24.4)	71 (24.7)	.**048**
Pregnancy	No	111	49 (14.4)	62 (21.6)	
	Unknown	362	208 (61.2)	154 (53.7)	
HIV status	Previously diagnosis	10	4 (1.2)	6 (2.1)	.**024**
	Negative	440	254 (74.7)	186 (64.8)	
	Unknown	177	82 (24.1)	95 (33.1)	
Past STIs	Chlamydia	113	62 (18.2)	51 (17.8)	.880
	Gonorrhea	70	42 (12.4)	28 (9.8)	.304
	Trichomonas	86	51 (15.0)	35 (12.2)	.309
	Syphilis	16	7 (2.1)	9 (3.1)	.394
	Herpes	27	18 (5.3)	9 (3.1)	.185
	HPV	28	18 (5.3)	10 (3.5)	.274
	Any STI above	218	122 (35.9)	105 (36.6)	.855
Symptoms	Lower abdominal pain	379	201 (59.1)	178 (62.0)	.459
	Vaginal bleeding	177	94 (27.7)	83 (28.9)	.724
	Vaginal discharge	175	88 (25.9)	87 (30.3)	.218
	Nausea or vomiting	157	77 (22.7)	80 (27.9)	.132
	Painful urination	110	53 (15.6)	57 (19.9)	.161
	Vaginal itching	60	38 (11.2)	22 (7.7)	.137
	Vaginal odor	27	14 (4.1)	13 (4.5)	.800
	Bumps, blisters, or sores	6	2 (0.6)	4 (1.4)	.420
	Pain during sex	7	1 (0.3)	6 (2.1)	.*052*
	Any symptom above	579	312 (91.8)	267 (93.0)	.552
Triage acuity	Level 1 (most acute)	13	8 (2.4)	5 (1.7)	.604
	Level 2	23	10 (2.9)	13 (4.5)	
	Level 3	255	145 (42.7)	110 (38.3)	
	Level 4	281	150 (44.1)	131 (45.6)	
	Level 5 (least acute)	55	27 (7.9)	28 (9.8)	
Pelvic examination	Performed	431	249 (73.2)	182 (63.4)	.**008**
	Not performed	196	91 (25.3)	105 (36.6)	
Abdominal computed tomography	Performed	118	64 (18.8)	54 (18.8)	.998
	Not performed	509	276 (81.2)	233 (81.2)	
Disposition	Discharge	525	286 (84.1)	239 (83.3)	.156
	Hospital observation	31	12 (3.5)	19 (6.6)	
	Admit to hospital	71	42 (12.4)	29 (10.1)	

^a^Central laboratory testing where a central laboratory tested for chlamydia (CT), gonorrhea (NG) with nucleic acid amplification test (NAAT), and for trichomonas (TV) with wet prep and/or NAAT based on provide order. ED POC testing where an ED POC laboratory tested for CT, NG, and TV with Visby Medical Sexual Health Test.

^b^italic *P* value indicating 0.1<*P*≤0.05; boldface P value indicating *P*<0.05.

### Chlamydia, Gonorrhea, and Trichomonas Testing

Of those 340 patients who received Central for CT and NG, 295 (86.8%) were tested for TV at the discretion of the ED provider (ie, an additional TV NAAT [n = 75] or wet prep test [n = 271] were ordered). All 287 patients in the ED POC group were tested for all three study STIs, as they are integrated on the POC device platform.

Overall, 78 (12.4%; 95% confidence interval [CI], 9.9–15.0) patients tested positive for one of the three STIs. For the 627 patients tested for CT and NG, 32 (5.1%) were positive for CT and 20 (3.2%) were positive for NG. For the 582 tested for TV, 42 (7.2%) were positive. There was a significantly higher TV positivity rate in those testing positive by ED POC versus those by Central wet prep test (ED POC: 9.4% vs wet prep: 3.7%. *P* = .007) but no significant differences in positivity for CT (ED POC: 5.6% vs Central: 4.7%, *P* = .622), NG (ED POC: 3.1% vs Central: 3.2%, *P* = .944), or Central TV NAAT (ED POC: 9.4% vs TV NAAT: 12.0%, *P* = .504). Furthermore, there were 14 patients (2.2%) coinfected with at least two study STIs, including five with both CT and NG, three with CT and TV, four with NG and TV, and two with all three.

### Antibiotic Treatment–based on Testing Approach

Overall, antibiotic undertreatment rates were low and there were no significant differences in undertreatment by test modality (Table 2). However, undertreatment rates for NG, TV (both patients received Central TV NAAT), as well as for CT/NG/TV in ED POC trended lower than those in Central (3.27 [95% CI, 0.44–24.34], 8.75 [95% CI, 0.45–171.17], 3.02 [95% CI, 0.84–10.82] times lower, respectively]. Among those with negative CT and NG results, rates of overtreatment were found to be significantly higher in the Central group for CT (Central: 11.1% vs ED POC: 3.0%; *P* < .001) and NG (Central: 10.6% vs ED-POC 4.0%; *P* = .002), but not for TV by Central wet prep test or TV NAAT (Central wet prep: 0.4% vs ED-POC: 0.8%; *P* = .997); Central TV NAAT: 3.0% versus ED POC: 0.8%; *P* = .367). Overall, those in the Central group were 2.8 times more likely to be treated unnecessarily for an STI than those in the ED POC group (Central: 11.0% vs ED-POC: 3.9%; *P* < .001).

**Table 2.** Undertreatment, Overtreatment, and Appropriate Treatment of 627 Female Emergency Department Patients With Sexually Transmitted Infection (STI) Testing for *Chlamydia trachomatis* (CT), *Neisseria gonorrhoeae* (NG), and *Trichomonas vaginalis* (TV) by Testing Modality and Individual STI

Antibiotic Treatment	STI	Overall	Central Lab Testing^d^	ED-POC Testing^d^	*P* Values^e^
		N = 627	N = 340	N = 287	
A. Undertreatment					
	CT positive	5/32 (15.6)	3/16 (18.8)	2/16 (12.5)	1.000
	NG positive	5/20 (25.0)	4/11 (36.4)	1/9 (11.1)	.319
	TV positive	2/42 (4.8)	2/15 (13.3)	0/27 (0.0)	.122
	Any positive	10/78 (12.8)	7/34 (20.6)	3/44 (6.8)	*.093*
B. Overtreatment					
	CT negative	44/595 (7.4)	36/324 (11.1)	8/271 (3.0)	**<.001**
	NG negative	46/607 (7.6)	35/329 (10.6)	11/278 (4.0)	**.002**
	TV negative	5/540^a^ (0.9)	3/280^a^ (1.1)	2/260 (0.8)	1.000
	Any negative^b^	48/624^b^ (7.7)	37/338^b^ (11.0)	11/286^b^ (3.9)	**<.001**
C. Appropriate					
	CT	578/627 (81.6)	301/340 (88.5)	277/287 (96.5)	**<.001**
	NG	576/627 (91.9)	301/340 (88.5)	275/287 (95.8)	**<.001**
	TV	575/582^a^ (98.8)	290/295^a^ (98.3)	285/287 (99.3)	.451
	All^c^	569/627 (90.8)	296/340 (87.1)	273/287 (95.1)	**<.001**

^a^45 patients in the laboratory-based NAAT/wet prep group did not have any testing for TV.

^b^The denominator of overtreatment—any negative excluded (1) 2 patients (1 in the laboratory-based NAAT/wet prep group and 1 in the POC PCR group) had all CT, NG, and TV positive and (2) 1 patient in the laboratory-based NAAT/wet prep group had both CT and NG positive but no TV testing.

^c^The numerator of “appropriate treatment—all” was the number of patients who were not undertreated and overtreated for any of STIs (CT, NG, or TV). “Proper treatment—all” for those 45 patients who did not receive any testing for TV was considered for the treatment for both CT and NG.

^d^Central laboratory testing where a central laboratory tested for chlamydia (CT), gonorrhea (NG) with nucleic acid amplification test (NAAT), and for trichomonas (TV) with wet prep and/or NAAT based on provide order. ED-POC testing whereby an ED POC laboratory tested for CT, NG, and TV with Visby Medical Sexual Health Test.

^e^ italitic *P* value indicating 0.1<*P*≤0.05; boldface *P* value indicating *P*<0.05.

### STI Result Notification and Subsequent ED Visits

Of those in the ED-POC group, 95.8% of STI results populated in the EMR before patient discharge, compared to only 2.9% of STI results for those tested by Central (*P* < .001). When comparing by test modality for those 78 STI-positive patients, patients in the ED POC group were more likely to be informed of their positive result (ED POC: 95.5% [42/44] versus Central: 61.8% [21/34]; *P* < .001) and more likely to receive their result in the ED prior to discharge (ED POC: 95.5% vs Central: 8.8%; *P* < .001). Informing positive TV result in the ED in the ED POC group (27/27, 100%) was significantly higher than those by TV NAAT (4/9, 44.4%) or wet prep (2/10, 20%) (*P* < .001 and *P* < .001, respectively). Overall, 19 (3.0%) patients had subsequent ED visits with STI-related chief complaints within 30 days after the index ED encounter (see Supplementary Results). We did not observe a significant difference in subsequent STI-related encounters by testing modality (Central: 1 [0.3%] versus ED POC: 2 [0.7%]; *P* = .873).

### Key Time Intervals of ED Workflow by Testing Modality

In the univariate analysis, there was a trend toward a significant difference in the overall unadjusted ED LOS between two groups (Central: 579 minutes (inerquartile range: 408.5–1020) versus ED POC: 536 minutes (interquartile range: 394–902), *P* = .085] (Table 3). In the multivariable analysis, ED POC significantly decreased ED LOS on average 9.3% (95% CI, 1.7–16.3) (ie, 76 minutes) after adjusting for triage acuity level, having abdominal computed tomography scan, and discharge from ED, three covariates that significantly affected the total ED LOS (Table 4). See Supplemental Results section for detailed bivariate and subgroup analysis.

**Table 3.** Comparisons of Key Time Intervals of Emergency Department Workflow Among Female Patients Who Received Laboratory-based Nucleic Acid Amplification Tests (NAAT) for Chlamydia, Gonorrhea, and Trichomonas and/or Wet Prep Test for Trichomonas and Those Who Received Point-of-Care Polymerase Chain Reaction (POC PCR) Test for Chlamydia, Gonorrhea, and Trichomonas in an Urban Academic Emergency Department, Baltimore, Maryland

	Median (Interquartile Interval) In M		
ED Workflow Time Interval	Central Lab Testing^a^	ED-POC Testing^a^	*P*-value^b^
All Patients	n = 340	n = 287	
Arrival to triage	4 (2, 8.5)	5 (2, 9)	.458
Triage to screening provider	64.5 (34.5, 117.5)	70 (31, 123)	.593
Screening provider to test order	160.5 (64.5, 314.5)	100 (9, 291)	**<.001**
Test order to specimen collection	40.5 (13.5, 95.5)	36 (16, 90)	.839
Specimen collection to resulting	1462 (1188, 2509.5)	47 (40, 59)	**<.001**
Room to discharge	393.5 (258.5, 630)	322 (205, 503)	**<.001**
Arrival to test order	269 (148, 488)	188 (96, 471)	**.003**
Arrival to specimen collection	347.5 (219.5, 559.5)	301 (156, 528)	**.002**
Arrival to resulting	1855.5 (1553.5, 2904.5)	351 (207, 597)	**<.001**
Arrival to discharge	579 (408.5, 1020)	536 (394, 902)	.*085*
Test order to discharge	293.5 (172.5, 470.5)	290 (184, 448)	.985
Specimen collection to discharge	220.5 (103.5, 387.5)	223 (113, 351)	.731

^a^Central laboratory testing where a central laboratory tested for chlamydia (CT), gonorrhea (NG) with nucleic acid amplification test (NAAT), and for trichomonas (TV) with wet prep and/or NAAT based on provide order. ED-POC testing where an ED POC laboratory tested for CT, NG, and TV with Visby Medical Sexual Health Test.

^b^italic *P* value indicating 0.1<*P*≤0.05; boldface *P* value indicating *P*<0.05.

**Table 4.** Multivariable Analysis on Time From Emergency Department (ED) Arrival to Departure in ED Patients Who Received STI Testing Order From 9 am to 12 am During the Study Period—All 627 Patients

Variables	Reference Group	Adjusted % Average LOS Increase (95% CI)	*P* Value^b^
Triage acuity—level 1 and 2	Increase each level of acuity	-17.2 (−21.8, −12.3)	**<.001**
Procedure—having abdominal CT scan	No abdominal CT scan	34.5 (20.4, 50.2)	**<.001**
ED POC testing^a^	Central lab testing^a^, no off-hours	−9.3 (−16.3, −1.7)	**.017**
Disposition—discharge from main ED	Admit or observation unit	−43.3 (−49.6, −36.2)	**<.001**

^a^Central lab testing whereby a central laboratory tested for chlamydia (CT), gonorrhea (NG) with nucleic acid amplification test (NAAT), and for trichomonas (TV) with wet prep and/or NAAT based on provide order. ED POC testing whereby an ED POC laboratory tested for CT, NG, and TV with Visby Medical Sexual Health Test.

^b^italic *P* value indicating 0.1<*P*≤0.05; boldface *P* value indicating *P*<0.05.

After adjusting for covariates, a sensitivity analysis that excluded the 43 patients who received Central in the ED POC integration phase did not change the overall finding that ED POC significantly decreased ED LOS on average 8.6% (95% CI, 1.7–16.3) (ie, 70 minutes) (Supplementary Table 8).

## DISCUSSION

Adoption of accurate POC diagnostics into clinical settings for evaluating patients with suspected STIs have been proposed to improve clinical care, yet limited implementation studies have been carried out to date. In this real-world integration of a recently FDA-cleared rapid POC PCR test for CT, NG, and TV, we observed significant reductions in rates of overtreatment for CT and NG from 11.1% and 10.6% to 3.0% and 4.0%, respectively. Although constrained by a small number of positive cases, we also observed a marginally improved rate of undertreatment for CT/NG/TV together, from 20.6% in central laboratory testing to 6.8% when transitioning to POC PCR testing. These findings reveal a significant opportunity to transform STI management from broad empirical treatment to precision-based, organism-specific therapy, thereby improving clinical outcomes and advancing antibiotic stewardship.

Over the past several decades, EDs have become a critical venue for management and care for individuals with suspected STIs in the United States. We found that 1 in 8 female patients with suspected STIs tested positive for at least one pathogen. Overall rates of STIs in the study were high, in line with prior studies in similar settings [9, 16, 17, 24]. Given the continuing surge of STIs and antimicrobial resistance in the United States [26, 27], and underserved populations disproportionately seeking care in the ED [28], the reliance on either a highly accurate STI testing but slow (1–2 day) turnaround test (ie, central laboratory NAAT testing) [12, 29], or rapid (30 minutes), but low sensitivity testing (ie, wet prep test) [24] poses pragmatic shortfalls that impact overall performance for both individual patient care, and ability to disrupt community transmission. Recent FDA clearance of highly accurate molecular POC tests for STIs [30] provides opportunities for more precision-based and timely management of ED patients with suspected STIs.

To address these challenges, effective integration of POC PCR testing for STIs into the ED workflow is vital to achieving the desired clinical outcomes, namely providing more targeted therapy for patients with suspected STIs, and decreasing rates of under- and overtreatment [31]. Accordingly, our study included developing and administering intensive education and training for ED clinical staff (both technicians and clinical providers), as well as streamlining the integration of an STI pathway guideline into the EMR before the implementation phase. Once implemented, our integrated POC PCR STI testing pathway delivered results to providers with a median turnaround time of 47 minutes (from specimen collection to results), which we believe represents the first successful integration of POC PCR testing for STIs into any ED.

Furthermore, when POC PCR was relied on, we observed significant improvement in positive-STI result notification in the ED. Notably, when Central was relied on, all seven undertreated patients were not informed of their positive result during their ED stay. This includes 4 (36%) of the 11 NG-positive patients who had no documented antibiotic treatment, despite follow-up attempts after discharge. Although we did observe a few cases of undertreatment for STIs when POC PCR testing was made available, these were likely preventable with further provider education (Supplemental Results Section).

We observed that use of POC PCR testing for STIs resulted in an approximately 10% reduction in patient's average LOS compared to central laboratory testing, after adjusting for key covariates and confounders. This translates into an approximately 80-minute average reduction in overall ED LOS for patients who received STI testing. Of note, we also observed that the interval from screening provider encounter to test ordering was 60 minutes longer when comparing central laboratory testing versus POC testing; this likely reflected to differences in workflow when POC was made available, and the tendency to defer ordering until later in the encounter when rapid results were not available. Earlier ordering in the POC group may have facilitated more timely clinical decision-making and contributed to greater efficiency in overall ED workflow. On the other hand, we observed no difference in the time interval between specimen collection time and discharge in the univariate analysis. One likely explanation for this observation is that when the provider's order central laboratory testing, their practice for STI management is based on syndromic practice (ie, test results are not available during the ED visit). In those cases, downstream decisions times are not heavily impacted. Thus, the principal driver for the observed overall reduction in time seen with POC testing are the fact that POC tests are being collected very early during the ED encounter. As described previously, this is also accompanied by significant reductions in overtreatment rates. Both cost analysis and cost-effectiveness analysis are under way to fully determine the potential utility of using POC PCR STI assays in the ED.

Our study has several limitations. First, our urban academic ED serves a socioeconomically vulnerable community with high incidence of bacterial STIs and established infrastructure for STI care, including onsite CLIA-waived POC testing, which may not be generalizable. Second, the observed results may be specific to our ED's provider practices, clinical workflow (ie, rapid screening providers ordering STI testing), crowding, and local STI epidemiology. Third, our ED POC laboratory did not operate overnight (12 am to 9 am), potentially underestimating the utility and full impact of POC PCR STI testing. Fourth, the significantly higher rate of TV infection observed when POC test is used, results from lack of sensitivity of traditional wet prep testing. Still, the real-world and favorable impact of POC testing remains (and is) applicable to testing practices for TV (ie, wet prep), which is now routinely used in EDs across the United States. Finally, we could not adjust for seasonal bias or differences in COVID-19 rates between the Central only phase and ED POC integration phase.

Our findings demonstrate that rapid, POC PCR testing for STIs can be effectively integrated into ED workflow, improving diagnostic timeliness and treatment fidelity (ie, significant reduction in overuse of antibiotics and a trend toward reducing rates of undertreatment) for female patients with suspected STIs. Use of a rapid (<30-minute turnaround time) multiplex molecular POC PCR could revolutionize ED management, leading to more precise, targeted treatment. Additionally, for patients receiving STI testing, the POC PCR testing approach used here led to a significant reduction in length of ED stay. Further cost and value analysis will be helpful for informing local practices and guiding dissemination of POC STI testing in EDs.

## Supplementary Material

ofaf749_Supplementary_Data

## Supplementary Data

Supplementary materials are available at *Open Forum Infectious Diseases* online. Consisting of data provided by the authors to benefit the reader, the posted materials are not copyedited and are the sole responsibility of the authors, so questions or comments should be addressed to the corresponding author.
