# Supplementary material for: Real-world Use of Molecular Point-of-care Testing for Sexually Transmitted Infections (STIs) in the Emergency Department: Why It Matters for Acute Care Management
Source: Open Forum Infect Dis. 2025 Dec 12;13(1):ofaf749. doi: 10.1093/ofid/ofaf749 (PMC12757586; doi:10.1093/ofid/ofaf749)
Supplement: ofaf749_Supplementary_Data [file ofaf749_supplementary_data.zip › OFID_Visby_SupplementTable_09252025.docx]

**Supplementary Tables**

**Supplement Table 1**. Socio-Demographic and Clinical Characteristics among 627 Female Johns Hopkins Hospital Adult Emergency Department (ED) Patients Who Received Testing for Chlamydia, Gonorrhea, and Trichomonas from August 1, 2022 to November 30, 2022 prior to ED Point-of-Care (ED-POC) Integration Phase and from January 9, 2023 to April 28, 2023 during ED-POC Integration Phase

|  |  | ED-POC Implementation | | | p-value |
| --- | --- | --- | --- | --- | --- |
| Characteristics | Category | Total | Central Only Phase^*^ | ED-POC Integration Phase^*^ |  |
| Overall |  | 627 | 297 | 330 |  |
| A. Socio-demographics | |  |  |  |  |
| Age (years) | Mean | 31.7±11.1 | 32.2±11.9 | 31.3±10.4 | 0.329 |
|  | Median | 30 [23, 36] | 29 [24, 36] | 30 [23, 37] | 0.657 |
|  | 18 – 24 | 193 | 87 (29.3) | 106 (32.1) | 0.265 |
|  | 25 – 29 | 115 | 64 (21.6) | 51 (15.5) |  |
|  | 30 – 34 | 126 | 55 (18.5) | 71 (21.5) |  |
|  | 35 – 39 | 80 | 34 (11.5) | 46 (13.9) |  |
|  | ≥ 40 | 112 | 56 (18.9) | 56 (17.0) |  |
|  | Missing | 1 | 1 ( 0.3) | 0 ( 0.0) |  |
| Gender | Female | 623 | 295 (99.3) | 328 (99.4) | 0.794 |
|  | Transgender Male | 3 | 1 ( 0.3) | 2 ( 0.6) |  |
|  | Non-Binary | 1 | 1 ( 0.3) | 0 ( 0.0) |  |
| Race/Ethnicity | White, Non-Hispanic | 68 | 35 (11.8) | 33 (10.0) | 0.874 |
|  | Black, Non-Hispanic | 403 | 191 (64.3) | 212 (64.2) |  |
|  | Other Race, Non-Hispanic | 54 | 25 ( 8.4) | 29 ( 8.8) |  |
|  | Hispanic | 102 | 46 (15.5) | 56 (17.0) |  |
| B. Clinical Characteristics | |  |  |  |  |
| Self-Reported | Yes | 154 | 69 (23.2) | 85 (25.8) | *0.056* |
| Pregnancy | No | 111 | 43 (14.5) | 68 (20.6) |  |
|  | Unknown | 362 | 185 (62.3) | 177 (53.6) |  |
| HIV Status | Previously Diagnosis | 10 | 4 ( 1.4) | 6 ( 1.8) | **0.039** |
|  | Negative | 440 | 223 (75.1) | 217 (65.8) |  |
|  | Unknown | 177 | 70 (23.6) | 107 (32.4) |  |
| Past STIs | Chlamydia | 113 | 59 (19.9) | 54 (16.4) | 0.255 |
|  | Gonorrhea | 70 | 41 (13.8) | 29 ( 8.8) | **0.046** |
|  | Trichomonas | 86 | 47 (15.8) | 39 (11.8) | 0.145 |
|  | Syphilis | 16 | 6 ( 2.2) | 10 ( 3.0) | 0.423 |
|  | Herpes | 27 | 18 ( 6.1) | 9 ( 2.7) | **0.040** |
|  | HPV | 28 | 16 ( 5.4) | 12 ( 3.6) | 0.289 |
|  | Any STI Above | 218 | 113 (38.1) | 114 (34.6) | 0.362 |
| Symptoms | Lower Abdominal Pain | 379 | 179 (60.3) | 200 (60.6) | 0.931 |
|  | Vaginal Bleeding | 177 | 80 (26.9) | 97 (29.4) | 0.495 |
|  | Vaginal Discharge | 175 | 84 (28.3) | 91 (27.6) | 0.844 |
|  | Nausea or Vomiting | 157 | 70 (23.6) | 87 (26.4) | 0.420 |
|  | Painful Urination | 110 | 49 (16.5) | 61 (18.5) | 0.514 |
|  | Vaginal Itching | 60 | 35 (11.8) | 25 ( 7.6) | *0.074* |
|  | Vaginal Odor | 27 | 13 ( 4.4) | 14 ( 4.2) | 0.934 |
|  | Bumps, Blisters or Sores | 6 | 1 ( 0.3) | 5 ( 1.5) | 0.220 |
|  | Pain During Sex | 7 | 1 ( 0.3) | 6 ( 1.8) | 0.127 |
|  | Any Symptom Above | 579 | 277 (93.3) | 302 (91.5) | 0.410 |
| Triage Acuity | Level 1 (most acute) | 13 | 7 ( 2.4) | 6 ( 1.8) | 0.709 |
|  | Level 2 | 23 | 8 ( 2.7) | 15 ( 4.6) |  |
|  | Level 3 | 255 | 118 (39.7) | 137 (41.5) |  |
|  | Level 4 | 281 | 138 (46.5) | 143 (43.3) |  |
|  | Level 5 (least acute) | 55 | 26 ( 8.8) | 29 ( 8.8) |  |
| Pelvic Exam | Performed | 431 | 222 (74.8) | 209 (63.3) | **0.002** |
|  | Not Performed | 196 | 75 (25.3) | 121 (36.7) |  |
| Abdominal CT | Performed | 118 | 53 (17.9) | 65 (19.7) | 0.554 |
|  | Not Performed | 509 | 244 (82.2) | 265 (80.3) |  |
| Disposition | Discharge | 525 | 257 (86.5) | 268 (81.2) | **0.017** |
|  | Hospital Observation | 31 | 7 ( 2.4) | 24 ( 7.3) |  |
|  | Admit to Hospital | 71 | 33 (11.1) | 38 (11.5) |  |

***** ‘Central Only Phase’ when only ‘Central Lab Testing’ operated from August 1 2022 to November 30 2022; ‘ED-POC Integration Phase’: the period when POC PCR testing was integrated with ED workflow from January 2023 to late April 2023

**Supplement Table 2**. Socio-Demographic Characteristics among 340 Female Johns Hopkins Hospital Adult Emergency Department (ED) Patients Who Received Central Lab Testing for Chlamydia, Gonorrhea, and Trichomonas from August 1, 2022 to November 30, 2022 prior to ED Point-of-Care (ED-POC) Integration Phase and from January 9, 2023 to April 28, 2023 during ED-POC Integration Phase

|  |  | ED-POC Implementation | | | p-value |
| --- | --- | --- | --- | --- | --- |
| Characteristics | Category | Total | Central Only Phase^*^ | ED-POC Integration Phase^*^ |  |
| Overall |  | 340 | 297 | 43 |  |
| Age (years) | Mean | 32.4±11.8 | 32.2±11.9 | 34.1±11.7 | 0.320 |
|  | Median | 30 [24, 36] | 29 [24, 36] | 32 [25, 40] | 0.194 |
|  | 18 – 24 | 97 | 87 (29.3) | 10 (23.3) | 0.744 |
|  | 25 – 29 | 71 | 64 (21.6) | 7 (16.3) |  |
|  | 30 – 34 | 63 | 55 (18.5) | 8 (18.6) |  |
|  | 35 – 39 | 41 | 34 (11.5) | 7 (16.3) |  |
|  | ≥ 40 | 67 | 56 (18.9) | 11 (25.6) |  |
|  | Missing | 1 | 1 ( 0.3) | 0 ( 0.0) |  |
| Gender | Female | 338 | 295 (99.3) | 43 (100) | 1.000 |
|  | Transgender | 1 | 1 ( 0.3) | 0 ( 0.0) |  |
|  | Non-Binary | 1 | 1 ( 0.3) | 0 ( 0.0) |  |
| Race/Ethnicity | White, Non-Hispanic | 41 | 35 (11.8) | 6 (14.0) | 0.563 |
|  | Black, Non-Hispanic | 215 | 191 (64.3) | 24 (55.8) |  |
|  | Other Race, Non-Hispanic | 28 | 25 ( 8.4) | 3 ( 7.0) |  |
|  | Hispanic | 56 | 46 (15.5) | 10 (23.3) |  |

***** ‘Central Only Phase’ when only ‘Central Lab Testing’ operated from August 1 2022 to November 30 2022; ‘ED-POC Integration Phase’: the period when POC PCR testing was integrated with ED workflow from January 2023 to late April 2023

**Supplement Table 3**: Bivariate Analysis on Factors Associated with Time from Emergency Department (ED) Arrival to Departure in 627 ED patients who received STI testing order from 9 am to 12 am during both study periods

|  |  |  |  |
| --- | --- | --- | --- |
| Variables | Reference Group | % Average LOS Increase (95% CI) | p-value |
|  |  |  |  |
| Age | Increase Each Year of Age | 0.8 ( 0.3, 1.3) | **<0.001** |
| Race – Minority (Black, Asian, or Other) | White | -16.9 (-28.3, -3.7) | **0.014** |
| Ethnicity - Hispanic | Non-Hispanic | 7.7 ( -5.5, 22.7) | 0.265 |
| HIV Status - Negative | Positive or Unknown | -9.1 (-18.1, 1.0) | *0.076* |
| Self-Reported Pregnancy | No Pregnancy or Unknown | -21.3 (-29.5, -12.1) | **<0.001** |
| Triage Acuity – Level 1 & 2 | Increase Each Level of Acuity | -26.0 (-30.4, -21.3) | **<0.001** |
| Symptom – Lower Abdominal Pain only | Other or No Symptom | 5.5 (-8.3, 21.3) | 0.456 |
| Symptom – Having Vaginal Discharge | Other or No Symptom | -9.8 (-18.9, 0.4) | *0.060* |
| Procedure – Having Pelvic Exam | No Pelvic Exam | -1.2 (-10.9, 9.7) | 0.824 |
| Procedure – Having Abdominal CT Scan | No Abdominal CT Scan | 75.2 (56.1, 96.5) | **<0.001** |
| ED-POC Testing | Central Lab Testing*, No Off-Hours | -9.6 (-17.9, -0.5) | **0.040** |
| STI Testing – CT, NG, or TV positive | No Positive Testing Result | -2.3 (-15.5, 13.1) | 0.757 |
| Disposition – Discharge from Main ED | Admit or Observation Unit | -53.6 (-58.7, -48.0) | **<0.001** |

* ‘Central Lab Testing’ whereby a central laboratory tested for chlamydia (CT), gonorrhea (NG) with nucleic acid amplification test (NAAT), and for trichomonas (TV) with wet prep and/or NAAT based on provide order. ‘ED-POC Testing’ whereby an ED POC laboratory tested for CT, NG, and TV with Visby Medical Sexual Health Test.

**Supplement Table 4:** Multivariable Regression Analysis on Time from Emergency Department (ED) Arrival to Departure in ED patients who received STI testing order from 9 am to 12 am during the study period by Triage Acuity

|  |  |  |  |
| --- | --- | --- | --- |
| Variables | Reference Group | Adjusted % Average LOS Increase (95% CI) | p-value |
|  |  |  |  |
| **Triage Acuity – Level 1, 2 & 3** |  |  |  |
| Self-Reported Pregnancy | No Self-Reported Pregnancy | -23.1 (-34.7, -9.5) | **0.002** |
| Procedure – Having Abdominal CT Scan | No Abdominal CT Scan | 21.2 ( 4.4, 40.6) | **<0.001** |
| ED-POC Testing^*^ | Central Lab Testing^*^, No Off-Hours | -15.6 (-25.5, -4.3) | **0.009** |
| Disposition – Discharge from Main ED | Admit or Observation Unit | -42.0 (-50.0, -32.7) | **<0.001** |
|  |  |  |  |
| **Triage Acuity – Level 4 & 5** |  |  |  |
| Self-Reported Pregnancy | No Self-Reported Pregnancy | 17.6 ( 4.9, 31.9) | **0.006** |
| Procedure – Having Abdominal CT Scan | No Abdominal CT Scan | 53.4 (28.5, 83.1) | **<0.001** |
| ED-POC Testing^*^ | Central Lab Testing^*^, No Off-Hours | -2.4 (-11.9, -8.2) | 0.646 |
| Disposition – Discharge from Main ED | Admit or Observation Unit | -42.1 (-52.9, -28.9) | **<0.001** |

* ‘Central Lab Testing’ whereby a central laboratory tested for chlamydia (CT), gonorrhea (NG) with nucleic acid amplification test (NAAT), and for trichomonas (TV) with wet prep and/or NAAT based on provide order. ‘ED-POC Testing’ whereby an ED POC laboratory tested for CT, NG, and TV with Visby Medical Sexual Health Test.

**Supplement Table 5:** Multivariable Regression Analysis on Time from Emergency Department (ED) Arrival to Departure in ED patients who received STI testing order from 9 am to 12 am during the study period by STI positivity

|  |  |  |  |
| --- | --- | --- | --- |
| Variables | Reference Group | Adjusted % Average LOS Increase (95% CI) | p-value |
|  |  |  |  |
| **STI Testing – Any Positive Results** |  |  |  |
| Triage Acuity – Level 1 & 2 | Increase Each Level of Acuity | -26.8 (-36.1, -16.1) | **<0.001** |
| ED-POC Testing^*^ | Central Lab Testing^*^, No Off-Hours | -15.5 (-31.3, 4.0) | 0.116 |
| Disposition – Discharge from Main ED | Admit or Observation Unit | -52.7 (-65.4, -35.3) | **<0.001** |
|  |  |  |  |
| **STI Testing – No Any Positive Results** |  |  |  |
| Self-Reported Pregnancy | No Self-Reported Pregnancy | -12.3 ( -2.8, -21.0) | **0.013** |
| Triage Acuity – Level 1 & 2 | Increase Each Level of Acuity | -16.2 (-21.3, -10.8) | **<0.001** |
| Procedure – Having Abdominal CT Scan | No Abdominal CT Scan | 31.6 ( 16.4, 48.7) | **<0.001** |
| ED-POC Testing^*^ | Central Lab Testing^*^, No Off-Hours | -8.2 (-15.8, 0.2) | *0.056* |
| Disposition – Discharge from Main ED | Admit or Observation Unit | -41.8 (-48.7, -33.9) | **<0.001** |

* ‘Central Lab Testing’ whereby a central laboratory tested for chlamydia (CT), gonorrhea (NG) with nucleic acid amplification test (NAAT), and for trichomonas (TV) with wet prep and/or NAAT based on provide order. ‘ED-POC Testing’ whereby an ED POC laboratory tested for CT, NG, and TV with Visby Medical Sexual Health Test.

**Supplement Table 6:** Multivariable Regression Analysis on Time from Emergency Department (ED) Arrival to Departure in ED patients who received STI testing order from 9 am to 12 am during the study period by ED disposition

|  |  |  |  |
| --- | --- | --- | --- |
| Variables | Reference Group | Adjusted % Average LOS Increase (95% CI) | p-value |
|  |  |  |  |
| **Discharge from ED** |  |  |  |
| Triage Acuity – Level 1 & 2 | Increase Each Level of Acuity | -18.3 (-23.1, -13.2) | **<0.001** |
| Procedure – Having Abdominal CT Scan | No Abdominal CT Scan | 35.4 ( 19.0, 54.1) | **<0.001** |
| ED-POC Testing^*^ | Central Lab Testing^*^, No Off-Hours | -7.3 (-14.8, 0.6) | *0.083* |
|  |  |  |  |
| **Admit to Hospital Obs. Unit/Floor** |  |  |  |
| Self-Reported Pregnancy | No Self-Reported Pregnancy | -46.4 (-62.8, -22.7) | **0.001** |
| ED-POC Testing^*^ | Central Lab Testing^*^, No Off-Hours | -11.6 (-29.6, 11.0) | 0.291 |

* ‘Central Lab Testing’ whereby a central laboratory tested for chlamydia (CT), gonorrhea (NG) with nucleic acid amplification test (NAAT), and for trichomonas (TV) with wet prep and/or NAAT based on provide order. ‘ED-POC Testing’ whereby an ED POC laboratory tested for CT, NG, and TV with Visby Medical Sexual Health Test.

**Supplement Table 7:** Multivariable Regression Analysis on Time from Emergency Department (ED) Arrival to Departure in ED Patients Who Received STI Testing Order from 9 Am to 12 Am during the Study Period by Presenting Symptoms – Lower Abdominal Pain Only

|  |  |  |  |
| --- | --- | --- | --- |
| Variables | Reference Group | Adjusted % Average LOS Increase (95% CI) | p-value |
|  |  |  |  |
| **Lower Abdominal Pain Only** |  |  |  |
| Triage Acuity – Level 1 & 2 | Increase Each Level of Acuity | -26.0 (-38.8, -10.5) | **0.036** |
| Self-Reported Pregnancy | No Self-Reported Pregnancy | -23.5 (-40.2, -2.2) | **0.003** |
| Procedure – Having Abdominal CT Scan | No Abdominal CT Scan | 29.7 ( 0.5, 67.2) | **0.049** |
| ED-POC Testing^*^ | Central Lab Testing^*^, No Off-Hours | -18.2 (-34.3, 1.7) | *0.075* |
| Disposition – Discharge from Main ED | Admit or Observation Unit | -34.1 (-51.0, -11.2) | **0.007** |
|  |  |  |  |
| **Not Lower Abdominal Pain Only** |  |  |  |
| Triage Acuity – Level 1 & 2 | Increase Each Level of Acuity | -16.0 (-21.0, -10.8) | **<0.001** |
| Procedure – Having Abdominal CT Scan | No Abdominal CT Scan | 33.6 ( 18.1, 51.2) | **<0.001** |
| ED-POC Testing^*^ | Central Lab Testing^*^, No Off-Hours | -7.5 (-15.2, 0.9) | *0.081* |
| Disposition – Discharge from Main ED | Admit or Observation Unit | -44.6 (-51.3, -37.1) | **<0.001** |

* ‘Central Lab Testing’ whereby a central laboratory tested for chlamydia (CT), gonorrhea (NG) with nucleic acid amplification test (NAAT), and for trichomonas (TV) with wet prep and/or NAAT based on provide order. ‘ED-POC Testing’ whereby an ED POC laboratory tested for CT, NG, and TV with Visby Medical Sexual Health Test.

**Supplement Table 8:** Multivariable Regression Analysis on Time from ED Arrival to Departure in ED patients who received STI testing order from 9 am to 12 am during the study period - Sensitivity Analysis on 584 patients including 297 patients who received Central Lab Testing (‘Central’) in the **‘**Central Only Phase**’**, and 287 who received ED-POC testing in the ‘ED-POC Integration Phase’

|  |  |  |  |
| --- | --- | --- | --- |
| Variables | Reference Group | Adjusted % Average LOS Increase (95% CI) | p-value |
|  |  |  |  |
| Triage Acuity – Level 1 & 2 | Increase Each Level of Acuity | -16.3 (-21.0, -11.3) | **<0.001** |
| Procedure – Having Abdominal CT Scan | No Abdominal CT Scan | 35.9 ( 21.3, 52.3) | **<0.001** |
| ED-POC Testing* | Central Lab Testing*, No Off-Hours | -8.6 (-15.7, -0.8) | **0.032** |
| Disposition – Discharge from Main ED | Admit or Observation Unit | -41.7 (-48.5, -34.0) | **<0.001** |
|  |  |  |  |

* ‘Central Lab Testing’ whereby a central laboratory tested for chlamydia (CT), gonorrhea (NG) with nucleic acid amplification test (NAAT), and for trichomonas (TV) with wet prep and/or NAAT based on provide order. ‘ED-POC Testing’ whereby an ED POC laboratory tested for CT, NG, and TV with Visby Medical Sexual Health Test.
