# Supplementary material for: Real-world Use of Molecular Point-of-care Testing for Sexually Transmitted Infections (STIs) in the Emergency Department: Why It Matters for Acute Care Management
Source: Open Forum Infect Dis. 2025 Dec 12;13(1):ofaf749. doi: 10.1093/ofid/ofaf749 (PMC12757586; doi:10.1093/ofid/ofaf749)
Supplement: ofaf749_Supplementary_Data [file ofaf749_supplementary_data.zip › OFID_Visby_Supplement_Methods_Results_09252025.docx]

**Supplementary Methods**

***STI Testing***

Standard-of-care (SOC) at our hospital for STI testing during the ‘Central Only Phase’ involved NAATs performed daily in batch in the central laboratory (Roche cobas® 6800 CT/NG assay and Roche cobas® 6800 TV/MG assay) on samples collected via urine, self-collected vaginal swab, or provider-collected cervical swab specimens, according to ED STI testing guidelines. For patients undergoing wet prep testing in the ED, a vaginal swab is collected either by the patient or a clinician, placing it into a tube containing 0.5 mL of sterile saline, and then transported to the central microbiology laboratory for processing. During the ‘ED-POC Integration Phase’, when ED providers ordered the POC STI test (made available during the initial screening exam), the patient was provided with printed and verbal instructions to self-collect a vaginal swab in the restroom. The vaginal swab specimen was then handed off to a nurse or clinical technician and transported immediately to the ED POC laboratory. Providers also had the option for vaginal swab specimens to be collected during a pelvic exam, if performed. Upon specimen arrival to the ED POC laboratory, trained certified nursing assistants (CNAs) immediately performed the Visby POC test following the manufacturer’s operational guidelines and quality control (QC) protocols, and manually entered results into the EMR.

***Integration of POC PCR STI Testing to ED Workflow***

POC PCR STI testing was integrated into ED workflows under the guidance of ED clinical administration (ED clinical director and nursing director) with support from Department of Pathology (POC Director) (Figure 1). Preparatory steps included institutional validation of the assay, updates of the EMR embedded ED STI testing guidelines, and integration of the test order into the EMR (EPIC). Prior to implementation of the POC testing, educational training was provided to ED physicians including basic information about the performance of the test, as well as practical aspects of testing (test ordering, specimen collection, interpretation of test results for providers). Tailored training specific for ED nursing and technician staff related to work flow was also provided for educating patients on self-swab procedures. Further, all CNAs who performed the test in the ED POC laboratory were trained in POC STI testing in compliance with pathology POC testing and QC guidelines. This POC STI testing initiative was under the direction of ED clinical leadership, with oversight from the Pathology department for QC compliance, given Joint Commission on Accreditation of Healthcare Organizations (JCAHCO) regulations. During the ‘ED-POC Integration Phase’, when the POC laboratory was operational from 9 am to midnight, all ED providers had access to order the POC STI panel as they deemed appropriate for female patients, including during the screening process and the initial patient work-up. During ED POC laboratory off-hours or when a provider chose to order traditional laboratory-based testing, previous SOC workflows remained in place for NAAT and wet prep testing.

***Data Collection and Analysis***

Demographic variables included age, race, ethnicity, sex at birth, and gender identity. Clinical characteristics included presenting symptoms, HIV status, pregnancy status, STI testing and results, disposition, ED visit timestamps, and patient follow-up information including result notification.

Bivariate analysis was performed to compare sociodemographic and clinical characteristics, under-, over-, and appropriate antibiotic treatment as well as ED workflow time interval variables between two testing modalities by using chi-square test or Fisher’s exact test for categorical data or non-parametric Wilcoxon rank sum test for continuous data. Subgroup analyses were performed by STI testing positivity, triage acuity level, lower abdominal pain as only presenting symptom, and ED disposition. A sensitivity analysis was performed after excluding patients who received ‘Central’ in the ‘ED-POC Integration Phase’.

**Supplementary Results**

**Characteristics of Patients Included**

Socio-demographic characteristics of patients based on implementation phase (**‘****Central Only Phase’ and** ‘**ED-POC Integration Phase’**) are summarized in Supplement Table 1. Overall, groups were similar with regard to sociodemographic characteristics (Table 1). In addition, regarding clinical and past medical historical, no significant differences were noted based on presence of STI-related symptoms and triage acuity except for having diagnosed with gonorrhea or herpes, self-reported pregnancy, ED disposition (Table 1). However, those in the ‘ED-POC Integration Phase’ were less likely to have had a pelvic exam (‘Central’: 74.8% vs. POC: 63.3%, p=0.002).

**Chlamydia, Gonorrhea, and Trichomonas Testing**

Of those tested by ‘Central’, there were a small number of cases where invalid tests were reported in the EMR, including 0.9% (3/340) for both CT and NG, and 4.0% (4/75) for TV (by NAAT). Of those tested by POC, 4.2% (12/287) resulted invalid on the first run. Upon re-run according to the POC manufacturer’s instructions, 0.3% (1/287) were reported as invalid in the EMR. Nevertheless, this patient received the NAAT test with valid results.

**Antibiotic Treatment Based on Testing Approach**

The antibiotic treatment rates by STI and testing modality group are summarized in Table 2. The overall proper treatment for the three study STIs together in the ‘ED-POC’ group was significantly higher than that in the ‘Central’ group (95.1% vs. 87.1%, p<0.001).

There were 3 cases of undertreatment for STIs (2 CT, 1 NG) even when POC PCR testing was made available. These included one case in which the provider discharged the patient prior to reviewing the positive test result available on EMR, and two in which the providers were attending to emergency acute care issues, preventing them from attending to the STI result prior to the patient being discharge. Of those 3 cases, 2 received adequate antibiotic treatment afterwards (one during their inpatient stay and shortly after at outpatient clinic) and 1 who was unable to be reached by follow-up calls by the clinical team never had a documented treatment noted.

Of the 7 patients who were undertreated in the ‘Central’ group, 2 (1 TV, 1 CT & TV) received proper antibiotics during an inpatient stay, 1 (NG) was discharged but returned to ED to receive proper treatment 2 days later, 4 (2 NG, 1 CT, and 1 CT& NG) did not have documented information regarding antibiotic treatment. Of the 3 patients who were undertreated in the ‘ED- POC’ group, 1 (CT & NG) was treated during an inpatient stay, 1 (CT) received treatment 3 weeks later at an Obstetrics & Gynecology clinic, and 1 (NG) was discharged prior to STI results and lost to follow up.

**STI Result Notification and Subsequent ED Visits**

These significant differences in delivering STI result were mostly driven by high informing rates of TV positive results in the ‘ED-POC’ group (ED-POC; 100% vs. Central: 26.7%, p<0.001). There was no significant difference in informing rates by test modality for CT and NG positive results (CT: ED-POC: 93.8% vs. Central: 81.3%, p=0.285, NG: ED-POC: 88.9% vs. Central: 90.9%, p=1.000).

Upon chart review of the 19 patients with subsequent ED visits with STI-related chief complaints within 30 days after the index ED encounter, 3 were STI-related encounters. One patient in the ‘Central’ group, did not receive empirical treatment in the ED due to being asymptomatic was called back for treatment for NG. One patient who came back 16 days later after receiving treatment for TV based on positive POC PCR result was likely to be re-infected by the partner who refused to be treated. The last patient who came in for dysuria, initially tested negative for all three STIs by POC PCR but received empirical treatment for CT and NG, returned 5 days later and tested positive for yeast infection by wet prep.

**Key Time Intervals of Emergency Department Workflow by Testing Modality**

Time intervals of the ED workflow sequence of patients undergoing STI testing including arrival to triage, triage to seen by screening provider, screening provider to STI test order, test order to specimen collection, specimen collection to receiving the result, room to discharge, and overall ED stay (i.e., arrival to discharge) as well as other key intervals including arrival to test order, arrival to specimen collection, arrival to receiving the result, test order to discharge, and specimen collection to discharge are summarized for all patients in Table 3. For workflow sequence of patients with STI testing, there were no differences in the length of time before being seen by a screening provider and from test order to specimen collection among 2 test modality groups (‘ED-POC’ versus ‘Central’). However, there were significantly shorter median times spent by patients in the ‘ED-POC’ group than the ‘Central’ group in the intervals between screening provider to test order, specimen collection to receiving the result, and room to discharge as well as arrival to test order, arrival to specimen collection, and arrival to receiving the result.

In bivariate analysis, non-white race, self-reported pregnancy, triage acuity of 1 or 2, disposition as being discharged from the main ED, and ‘ED-POC’ significantly decreased total length of ED stay by 16.9%, 21.3%, 26.0%, 53.6%, and 9.6%, respectively, as compared to their counterpart reference group (Supplement Table 2). On the other hand, increasing age, and having an abdominal CT scan performed in the ED significantly increased total length of ED stay by 0.8% and 75.2%, respectively.

Subgroup analyses showed that ‘ED-POC’ significantly decreased ED LOS 15.6% (95%CI: 4.3%, 25.5%) in those with higher triage acuity (1 to 3) and marginally decreased 7.3% (95%CI: -1.0%, 14.8%) in patients who were discharged, 8.2% (95%CI: -0.2%, 15.8%) in STI negative patients, and 18.2% (95%CI: -1.7%, 34.3%) in those with a symptom of lower abdominal pain only (Supplement Tables 4-7).
